# Brown adipose tissue influences adiponectin and thyroid hormone changes during Graves’ disease therapy

**DOI:** 10.1080/21623945.2022.2104509

**Published:** 2022-07-27

**Authors:** Wei-En Ho, Lijuan Sun, Hui Jen Goh, Mya Thway Tint, Lei Sun, Melvin Khee Shing Leow

**Affiliations:** a Lee Kong Chian School of Medicine, Nanyang Technological University (NTU), Singapore, Singapore; b Singapore Institute for Clinical Sciences, Agency for Science, Technology and Research (A *STAR), Singapore; c Human Potential Translational Research Programme, Yong Loo Lin School of Medicine, National University of Singapore, Singapore; d Cardiovascular and Metabolic Disorders Program, Duke-NUS Medical School, Singapore; e Department of Endocrinology, Division of Medicine, Tan Tock Seng Hospital (TTSH), Singapore

**Keywords:** hyperthyroidism, transition, adiponectin, brown adipose tissue, thyroid hormone

## Abstract

Thyroid hormones (TH), adiponectin and brown adipose tissue (BAT) are regulators of energy homoeostasis. Influence of BAT activity on the relationship between TH and adiponectin remains unexplored. The aim of the study was to identify the relationship between TH and adiponectin and to clarify the impact of active BAT on the metabolic effects of adiponectin before and after the correction of thyrotoxicosis. Twenty-one patients with newly diagnosed hyperthyroidism from Graves’ disease were recruited. A titration dosing regimen of thionamide anti-thyroid drug (ATD) was used to establish euthyroidism over 12–24 weeks. Anthropometric, biochemical and adipocytokine parameters were measured before and after control of hyperthyroidism. BAT activity was quantified by fusion 18 F-fluorodeoxyglucose (18 F-FDG) PET/MR imaging, and patients were grouped based on BAT status. Plasma adiponectin level was significantly increased following correction of hyperthyroidism in the overall sample. Free thyroxine (FT4) was also identified as a predictor of adiponectin level in thyroid dysfunction. However, significant changes in adiponectin level and correlations involving adiponectin were absent in BAT-positive patients but maintained in BAT-negative patients. BAT activity diminishes the correlative relationship with body composition and abolishes TH and adiponectin relationships when transitioning from a hyperthyroid to euthyroid state.

## 1. Introduction

Hyperthyroidism leads to a hypermetabolic state, resulting in patients losing weight despite an increased appetite and caloric intake. The mechanism of thyroid hormones (TH), measured by plasma-free thyroxine (FT4) and free triiodothyronine (FT3), involvement in human metabolism has been widely explored, with TH found to increase lipid and carbohydrate metabolism as well as insulin resistance [1–5]. Adiponectin, an adipocytokine synthesized by adipocytes, has been shown to possess metabolically beneficial functions, serving a cardioprotective role as an anti-atherogenic and anti-inflammatory mediator as well as an insulin sensitizer [6–10]. Both TH and adiponectin play crucial roles in metabolic regulation, with both having an effect on energy homoeostasis, body temperature and basal metabolic rate. Studies done to establish adiponectin’s relationship to thyroid hormones have shown that shifts in serum levels of either causes fluctuations in the other, though findings have been mixed [11–14]. In hyperthyroid patients, the clear understanding as to whether adiponectin contributes to or offsets the effects of excessive TH may serve to further advance clinical treatment of hyperthyroid conditions such as Graves’ disease. As the biological significance of adiponectin levels in thyrotoxicosis remains obscure, the importance of dissecting the causal nexus between TH and adiponectin continues to be a research priority.

Brown adipose tissue (BAT) is another vital player in energy homoeostasis and thermogenesis in humans, contributing to 2.5–5% of the Resting Metabolic Rate (RMR) [15–17]. Hyperthyroidism is known to increase brown fat activity, through the effects of TH on the browning of white adipose tissue (WAT) [18]. Little is known about the influence of BAT activity on the relationship between TH and adiponectin.

Hence, our study aims to explore potential advancements in the treatment of Graves’ disease by delving deeper into this relationship between TH and adiponectin and providing a fuller picture by considering other metabolic factors such as BAT activity.

## 2. Materials and methods

### 2.1. Study participants

Participants with newly diagnosed Graves’ disease were enrolled from the endocrine outpatient clinics of a local tertiary hospital. Diagnostic criteria for Graves’ disease were based on clinical and biochemical evidence of primary hyperthyroidism and a positive plasma TSH receptor autoantibody test. Exclusion criteria included pregnancy, allergies to antithyroid drugs (ATD) (carbimazole/thiamazole) or a history of claustrophobia that hindered magnetic resonance (MR) imaging. Patients taking medications that could potentially affect anthropometric measurements (e.g. steroids) or BAT activity (e.g. beta-blockers) were also excluded.

### 2.2. Study protocol

**Figure 1.**
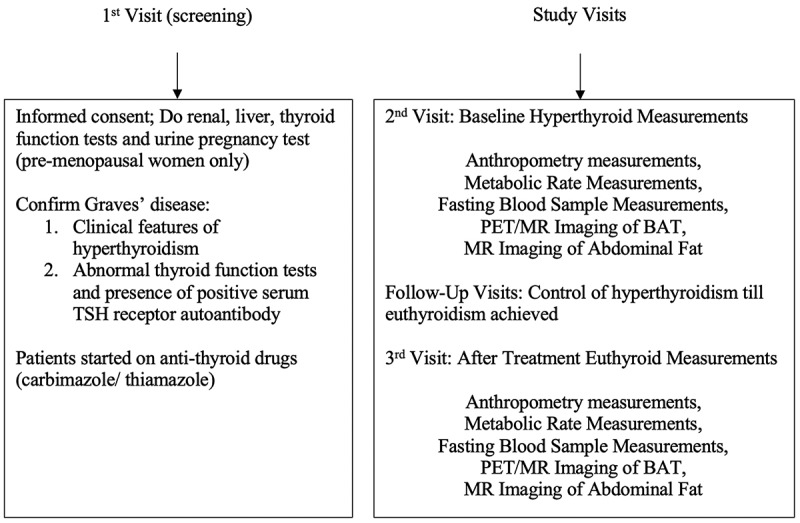
Study protocol.

The study protocol can be seen in Figure 1. Over three separate study visits, patients were screened, and pre- and post-treatment measurements were collected. During follow-up visits, patients were treated with a decremental dosing regimen of ATD with either carbimazole or thiamazole over 12–24 weeks. ATD doses were titrated against clinical symptoms and TFTs until euthyroidism was achieved.

Measurements were made at the Clinical Nutrition Research Centre (CNRC), and each measurement was preceded by an overnight fast. Anthropometry measurements (i.e. body composition) were measured by dual-energy X-ray absorptiometry (DXA), and energy expenditure data (i.e. resting metabolic rate (RMR)) was measured with infrared thermography (IRT) in a whole-body room calorimeter (WBC) for 45 minutes. Fasting blood samples were taken and sent to the National University Hospital Singapore (NUHS) referral laboratory for biochemical analyses. All positron emission tomography (PET) and magnetic resonance (MR) scans were performed at the Clinical Imaging Research Center (CIRC). An infusion of radio-labelled glucose marker (18 F-FDG) was used for PET/MR scanning for brown fat. Details of the operator settings used for our clinical measurements can be found in our previous publication [19].

### 2.3. Categorizing patients by BAT status

BAT status was determined using PET/MR scanning (Biograph mMR, Siemens Healthcare, Erlangen, Germany) for brown fat. Following 18 F-FDG infusion, a graph cut algorithm (ISMRM fat-water toolbox) was used for fat-water separation and ITK-SNAP was used to manually segment the supraclavicular BAT (sBAT) depots. These were done under close guidance of an experienced clinical radiologist. A threshold of 40% of supraclavicular fat fraction (sFF) values was used to exclude muscle and bone before the final values of MR sFF, PET standardized uptake value (SUV) mean and PET SUV max were derived. The cut-off value of sBAT PET SUV max for categorizing subjects into BAT-positive and BAT-negative groups was 1.5 [19].

### 2.4. Statistical analyses

Statistical analysis was performed by using SPSS Statistics Version 28 (IBM SPSS Inc.). Paired t-tests were used to identify significant changes following treatment. Pearson correlation was used to evaluate any potential relationships among variables. After separating patients based on BAT status, Student's t-test was used to compare differences between the two subgroups. All data are presented as mean ± standard error of the mean (SEM), with P-value of ≤0.05 considered statistically significant.

## 3. Results

### 3.1. Overall patient sample (n = 21)

#### 3.1.1. Characteristics of the patients following treatment of hyperthyroidism

The treatment results of the overall sample are shown in Table 1. Mean age of the participants was 39.5 ± 2.5 years. There was a significant increase in body weight, body mass index (BMI), RMR, lean and fat mass from hyperthyroidism to euthyroidism. With regard to BAT activity, only a significant increase in sFF mean was noted.

**Table 1.** Clinical and anthropometric patient data and summary of treatment outcomes.

Clinical and anthropometric parameters (n = 21)			
Parameter	Hyperthyroid	Euthyroid	*P*-value
	Mean ± SEM	Mean ± SEM	
Weight (kg)	55.6 ± 2.6	58.5 ± 2.9	**0.001**
BMI (kg/m^2^)	21.7 ± 0.9	22.9 ± 1.1	**0.001**
Fat mass (kg)	19.5 ± 1.6	20.3 ± 1.7	**0.040**
Lean mass (kg)	33.8 ± 1.6	35.7 ± 1.8	**<0.001**
Waist circumference (cm)	77.5 ± 2.7	80.4 ± 2.6	0.055
Mean SUV (g/mL)	0.9 ± 0.1	0.8 ± 0.1	0.926
Max SUV (g/mL)	1.7 ± 0.2	1.9 ± 0.2	0.518
sFF mean (%)	72.3 ± 1.4	76.8 ± 1.4	**<0.001**
Free T4 (pmol/L)	29.9 ± 3.0	12.3 ± 1.3	**<0.001**
Free T3 (pmol/L)	10.5 ± 0.9	5.5 ± 0.3	**<0.001**
Glucose (mmol/L)	4.4 ± 0.1	4.5 ± 0.1	0.714
Albumin (g/L)	40.2 ± 0.6	43.4 ± 0.5	**<0.001**
Cholesterol (mmol/L)	4.6 ± 0.2	5.6 ± 0.3	**<0.001**
Triglyceride (mmol/L)	1.4 ± 0.2	1.2 ± 0.2	0.060
HDL-C (mmol/L)	1.4 ± 0.1	1.7 ± 0.1	**<0.001**
LDL-C (mmol/L)	2.6 ± 0.2	3.3 ± 0.2	**0.001**
Insulin (mU/L)	5.4 ± 0.7	6.1 ± 0.8	0.330
HOMA-IR score	1.1 ± 0.1	1.2 ± 0.2	0.388
RMR (kcal/d)	1718.6 ± 87.7	1430.2 ± 65.2	**<0.001**
SAT (cm^3^)	54.7 ± 5.9	53.9 ± 5.6	0.210
VAT (cm^3^)	22.9 ± 2.9	22.6 ± 3.1	0.681
DSAT (cm^3^)	31.8 ± 4.2	30.2 ± 4.0	0.898
SSAT (cm^3^)	22.9 ± 1.8	23.7 ± 1.7	**0.007**
Adiponectin (ng/mL)	5981.0 ± 832.1	7800.7 ± 793.6	**0.001**

Data presented as means ± SEM. P-values represent paired t-tests between patient measurements at baseline hyperthyroid state and achievement of euthyroidism after treatment.

Serum total cholesterol, high-density lipoprotein cholesterol (HDL-C), and low-density lipoprotein cholesterol (LDL-C) increased significantly, while intriguingly, there were no alterations in serum glucose, insulin, or the Homoeostatic Model Assessment for Insulin Resistance (HOMA-IR) scores. Apart from superficial subcutaneous adipose tissue (SSAT) (P = 0.007), no other significant differences were noted in white adipose tissue (WAT) markers. Adiponectin increased significantly while transitioning from hyperthyroidism to euthyroidism.

#### 3.1.2. Relationship of adiponectin and body composition

**Figure 2.**
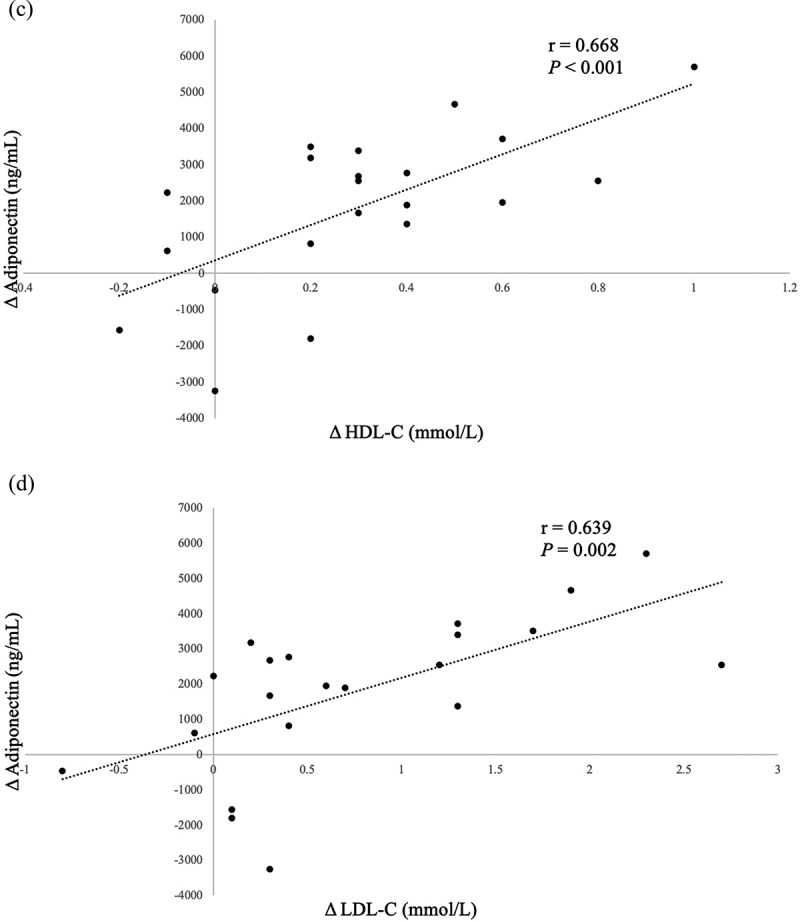
(continued).

In the overall sample, we noted significant correlations between changes in FT4 (r = −0.562, P = 0.008), cholesterol (r = 0.719, P < 0.001) levels, HDL-C (r = 0.668, P < 0.001), LDL-C (r = 0.639, P = 0.002) and changes in adiponectin levels. These findings are represented in Figure 2. No significant associations involving BMI, waist circumference, WAT markers or subcutaneous adipose tissue to visceral adipose tissue ratio (SAT:VAT) were noted.
**Figure 2.**
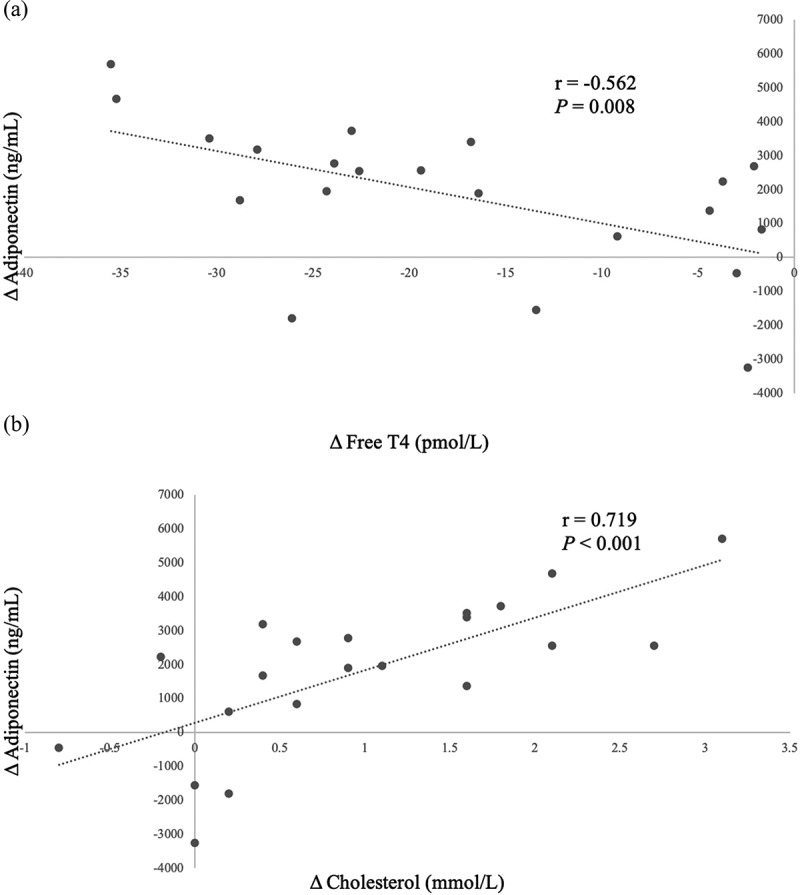
Significant Pearson correlations between Δ adiponectin (ng/mL) and (a) Δ FT4, (b) Δ serum cholesterol, (c) Δ HDL-C, (d) Δ LDL-C following correction of hyperthyroidism. These markers thus serve as good predictors for serum adiponectin changes during the correction of hyperthyroidism in patients with Graves’ disease.

### 3.2. BAT-positive (n = 8) and BAT-negative (n = 13) subgroups

#### 3.2.1. Characteristics of BAT-positive and BAT-negative patients

**Figure 3.**
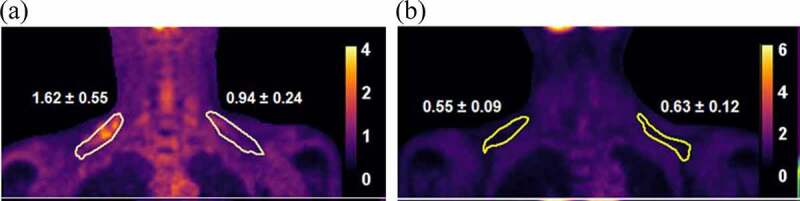
Representative 18 F-FDG PET images illustrating tracer uptake and standardized uptake values (SUV) within the circumscribed regions of interest (ROI) correlating to supraclavicular brown adipose tissue (sBAT) depots. There is higher tracer uptake and SUV in a (a) BAT-positive hyperthyroid patient and a correspondingly lower uptake and SUV within the same ROI in a (b) BAT-negative hyperthyroid patient.

The 21 subjects were then subdivided based on BAT status (Table 2). The cut-off value of max SUV for grouping the patients was 1.5 [20,21]. 18 F-FDG images of both subgroups in Figure 3 clearly depict the differences in the BAT-positive and BAT-negative patients. As expected, differences in measurements for BAT activity were significant between both groups. There was a significantly higher baseline FT4 level in the BAT-positive group. No significant differences in measured adiponectin levels at baseline, after treatment or degree of change were noted between the groups (Table 2).

**Table 2.** Significant differences in measured variables and adiponectin measurements between BAT-positive and BAT-negative patients.

Parameter	BAT-positive (n = 8)	BAT-negative (n = 13)	*P*-value
	Mean ± SEM	Mean ± SEM	
Mean SUV HT (g/mL)	1.2 ± 0.1	0.7 ± 0.1	**0.011**
Max SUV HT (g/mL)	2.4 ± 0.3	1.3 ± 0.1	**0.014**
sFF mean HT (%)	68.1 ± 1.5	74.9 ± 1.8	**0.010**
Glucose HT (mmol/L)	4.7 ± 0.1	4.2 ± 0.1	**0.042**
Cholesterol HT (mmol/L)	4.0 ± 0.3	5.0 ± 0.3	**0.045**
LDL-C HT (mmol/L)	2.1 ± 0.2	2.9 ± 0.3	**0.049**
Free T4 HT (pmol/L)	37.9 ± 4.6	25.0 ± 3.3	**0.038**
sFF mean ET (%)	72.7 ± 1.1	79.3 ± 1.9	**0.007**
Triglyceride ET (mmol/L)	0.7 ± 0.1	1.5 ± 0.3	**0.008**
Insulin ET (mU/L)	4.3 ± 0.7	7.3 ± 1.2	**0.040**
HOMA-IR ET	0.8 ± 0.1	1.5 ± 0.2	**0.037**
Free T4 ET (pmol/L)	−25.1 ± 3.9	−13.0 ± 2.6	**0.023**
Adiponectin HT (ng/mL)	7211.2 ± 1441.5	5223.9 ± 996.4	0.277
Adiponectin ET (ng/mL)	9410.5 ± 1290.0	6810.1 ± 940.0	0.125
Δ Adiponectin (ng/mL)	2199.4 ± 957.9	1586.2 ± 516.9	0.584

Data presented as mean ± SEM. P values represents Student’s t-test done to identify differences in variables based on BAT status. Highlighted cells point out the absence of any significant difference in adiponectin measurements between the groups.

#### 3.2.2. Differences in outcomes of treatment between BAT-positive and BAT-negative patients

When the outcomes of treatment of both groups were separately analysed (Table 3), the significant changes noted in weight, BMI, fat mass, lean mass, cholesterol, SSAT and adiponectin in the overall sample were preserved in the BAT-negative group. However, in the BAT-positive group, these significant changes were lost upon treatment. Although the increase in HDL-C value is the same in both groups (1.4 to 1.7 mmol/L), this increase was statistically insignificant in the BAT-positive group. We believe this is likely attributed to the smaller subgroup size of the BAT-positive patients.

**Table 3.** Differences in treatment outcomes of BAT-positive and BAT-negative patients.

Parameter	BAT-positive (n = 8)			BAT-negative (n = 13)		
	Hyperthyroid	Euthyroid	*P*-value	Hyperthyroid	Euthyroid	*P*-value
	Mean ± SEM	Mean ± SEM		Mean ± SEM	Mean ± SEM	
sFF mean (kg)	68.1 ± 1.3	72.7 ± 1.1	**0.011**	74.9 ± 1.8	79.3 ± 1.9	**<0.001**
Weight (kg)	52.4 ± 3.3	53.9 ± 3.6	0.088	57.6 ± 3.5	61.4 ± 4.1	**0.004**
BMI (kg/m^2^)	20.5 ± 0.8	21.1 ± 0.8	0.100	22.4 ± 1.3	24.0 ± 1.6	**0.003**
Fat mass (kg)	17.2 ± 1.5	17.3 ± 1.7	0.772	20.9 ± 2.4	22.2 ± 2.4	**0.036**
Lean mass (kg)	32.9 ± 2.4	34.1 ± 2.5	0.123	34.3 ± 2.0	36.7 ± 2.6	**0.002**
Cholesterol (mmol/L)	4.0 ± 0.3	4.9 ± 0.4	0.061	5.0 ± 0.3	6.0 ± 0.4	**0.002**
HDL-C (mmol/L)	1.4 ± 0.1	1.7 ± 0.2	0.074	1.4 ± 0.1	1.7 ± 0.1	**0.001**
LDL (mmol/L)	2.1 ± 0.2	2.9 ± 0.3	**0.045**	2.9 ± 0.3	3.6 ± 0.3	**0.009**
FT4 (pmol/L)	37.9 ± 4.1	12.8 ± 1.7	**<0.001**	25.0 ± 3.3	11.9 ± 1.9	**<0.001**
FT3 (pmol/L)	13.0 ± 1.5	5.9 ± 0.6	**0.002**	8.9 ± 0.9	5.2 ± 0.3	**<0.001**
RMR (kcal/d)	1839.0 ± 150.4	1493.7 ± 150.5	**0.006**	1644.5 ± 95.2	1391.1 ± 54.7	**<0.001**
SSAT (cm^3^)	21.3 ± 2.1	22.0 ± 2.2	0.228	22.9 ± 2.5	24.8 ± 2.5	**0.018**
Adiponectin (ng/mL)	7211.2 ± 1271.3	9410.5 ± 1290.0	0.055	5223.9 ± 996.4	6810.1 ± 940.0	**0.010**

Values are presented as mean ± SEM. P-values represent paired t-tests between BAT-positive and BAT-negative patient measurements at baseline hyperthyroid state and achievement of euthyroidism after treatment respectively. Highlighted cells point out differences in post-treatment outcomes between BAT subgroups.

**Figure 4.**
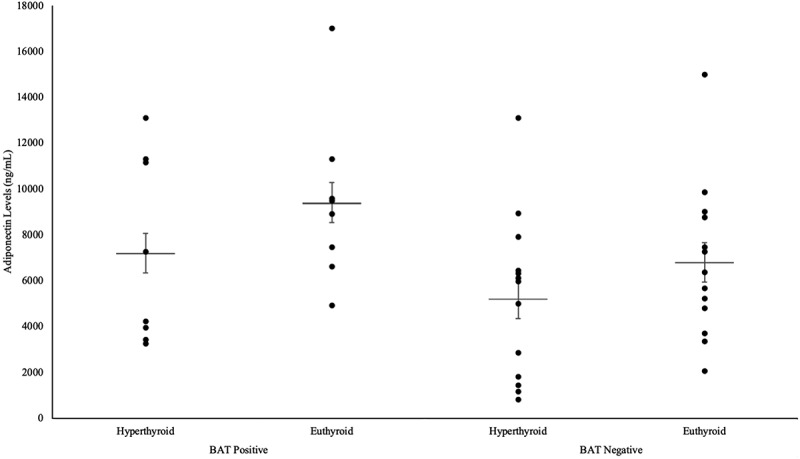
Differences in adiponectin levels in hyperthyroid and euthyroid states in both BAT-positive and BAT-negative groups.

As illustrated in Figure 4, the increase in adiponectin levels in the BAT-positive group from 7211.2 ± 1271.3 ng/mL to 9410.5 ± 1290.0 ng/mL was of a higher value than the adiponectin increase in the BAT-negative group, from 5223.9 ± 996.4 ng/mL to 6810.1 ± 940.0 ng/mL. However, only the increase in adiponectin in the BAT-negative group was deemed to be statistically significant (P = 0.010). This is due to the BAT-negative patients having lower adiponectin values at baseline, resulting in a higher magnitude of change after treatment.

Remarkably, we noted that the associations between adiponectin and FT4 differed between subgroups (Table 4). In the BAT-negative group, the significant association involving FT4 and adiponectin was maintained from the overall sample (P = 0.017). However, in the BAT-positive group, FT4 remarkably lost its association with adiponectin (P = 0.189).

**Table 4.** Loss of FT4 association with adiponectin in BAT-positive patients.

Parameter	Overall (n = 21)		BAT-positive (n = 8)		BAT-negative (n = 13)	
	Δ Adiponectin (ng/mL)		Δ Adiponectin (ng/mL)		Δ Adiponectin (ng/mL)	
	Coefficient	*P*-value	Coefficient	*P*-value	Coefficient	*P*-value
Δ Albumin (mmol/L)	0.633	**0.002**	0.74	**0.036**	0.633	**0.02**
Δ Cholesterol (mmol/L)	0.719	**<0.001**	0.781	**0.022**	0.684	**0.01**
Δ HDL-C (mmol/L)	0.668	**<0.001**	0.71	**0.049**	0.672	**0.012**
Δ LDL-C (mmol/L)	0.639	**0.002**	0.778	**0.023**	0.516	0.071
Δ HOMA-IR score	−0.409	0.066	−0.305	0.462	−0.484	0.094
Δ Free T4 (pmol/L)	−0.562	**0.008**	−0.517	0.189	−0.648	**0.017**

Pearson Correlations were used to assess relationships between variables. Highlighted cells point out differences in correlations between BAT subgroups.

**Figure 5.**
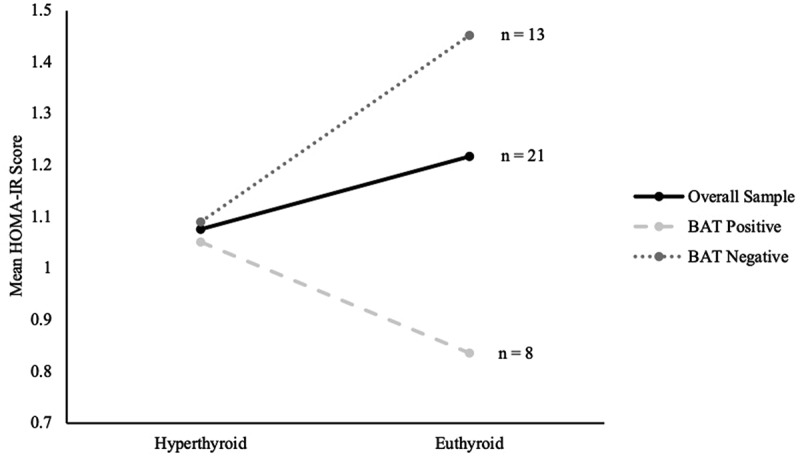
Changes in mean HOMA-IR scores between hyperthyroid and euthyroid states of the overall sample, BAT-positive and BAT-negative subjects.

Figure 5 reflects the changes in HOMA-IR scores between hyperthyroid and euthyroid states for the overall sample and both subgroups. In the overall sample, HOMA-IR scores increased from 1.07 ± 0.1 to 1.22 ± 0.2 following correction of hyperthyroidism. In the BAT-positive subjects, HOMA-IR scores decreased from 1.05 ± 0.2 to 0.84 ± 0.1 and in the BAT-negative group, HOMA-IR scores increased from 1.09 ± 0.2 to 1.45 ± 0.2. HOMA-IR scores showed no statistically significant changes following correction of hyperthyroidism in the overall sample (P = 0.388), and this was similar in both BAT-positive (P = 0.242) and BAT-negative (P = 0.130) groups. However, it is interesting to note that patients with active BAT-activity had lower HOMA-IR scores at the end of treatment despite similar scores at the baseline. Adiponectin did not have any significant associations with HOMA-IR scores (Table 4) in the overall sample (P = 0.066), as well as both BAT-positive (P = 0.462) and BAT-negative (P = 0.094) groups.

## 4. Discussion

Graves’ disease is an autoimmune thyroid disease commonly presenting as primary hyperthyroidism (rarely as euthyroid or hypothyroid Graves’ disease due to high levels of thyroid-stimulating blocking antibodies) resulting in alterations of body composition, insulin sensitivity, adiponectin levels and BAT function [6–10,15–18]. In our current study, the interplay of relationships between TH, adiponectin and BAT can be summarized by our key findings: 1) adiponectin level was lower in a hyperthyroid state, 2) FT4 is a predictor for adiponectin level and 3) BAT activity protects against significant metabolic changes in the correction of thyrotoxicosis.

From our data, there were noticeable alterations in body composition, energy expenditure and adipokines from hyperthyroid to euthyroid state. Weight and BMI, along with both fat and lean mass were noted to have increased significantly post-treatment. Following the attainment of euthyroidism, the rise in lean mass is due to decreased protein turnover [22], while increase in fat mass is accounted for by the significant fall of energy expenditure [23]. Adipokine changes were also noted, as shown by a rise in adiponectin following the correction of hyperthyroidism. Adjustments done for BMI, age, gender and ethnicity all showed lower adiponectin during hyperthyroidism. Various studies have conflicting findings regarding the effects of excessive TH on serum adiponectin, with some animal experimental studies reporting increased adiponectin [13,24], and other human studies reporting unchanged [25,26] or increased [27,28] adiponectin concentrations in hyperthyroidism. However, in line with Hsieh et al. [29] and a local study done by Chng et al. [30], our data showed lower adiponectin levels in TH excess, which increased following attainment of euthyroidism. A potential explanation for this finding suggested by Chu et al. was that the state of hyperthyroidism alters the neurophysiology of food regulation, hence driving marked hyperphagia and carbohydrate craving. A diet high in glycaemic load is known to result in decreased adiponectin levels [31]. Another explanation may involve the paradoxically inverse relationship between adiponectin and insulin resistance, as numerous studies have consistently found diminished adiponectin levels in obese individuals and patients with type 2 diabetes mellitus [7–10]. Graves’ thyrotoxicosis is a condition believed to lead to an insulin-resistant state [1,2], and hence adiponectin levels would theoretically be lower in hyperthyroidism. Overall, the effect of thyrotoxicosis on adiponectin still merits more mechanistic studies.

Next, we explored the use of various parameters in predicting for serum adiponectin changes during the correction of hyperthyroidism. Firstly, FT4 changes was found to have a negative correlation with serum adiponectin changes. This inverse relationship was demonstrated by Yaturu et al. [27] and Hsieh et al. [29], who both showed that TH, specifically FT4, might influence circulating levels of adiponectin when transitioning from hyperthyroidism to euthyroidism. Fernandez-Real et al. also identified serum FT4 to be a predictive variable of adiponectin in healthy subjects, and they suggested that TH acts as a regulator of adiponectin secretion [32]. Notably, serum cholesterol, HDL-C and LDL-C were all positively correlated with adiponectin levels. Adiponectin has a cardioprotective role [6] and stimulates PPAR-α activity, which increases HDL-C production and mitochondrial fatty acid beta oxidation [33,34]. Between adiponectin and LDL-C, a systemic review suggested an inverse relationship between the two [35], and our discordant finding remains unexplained. This positive correlation between adiponectin and LDL-C appears more striking for the BAT-negative group, as the increase in LDL-C was not as large in the BAT-positive group compared to the BAT-negative group. Other studies have reported BMI, waist circumference, WAT parameters and adiposity distribution (SAT:VAT ratio) to be strong predictors for adiponectin [6,29,36,37], but these markers did not yield any significant associations in our present study. Comparing the degree of correlations from our data, serum cholesterol levels seem to be the strongest predictor of adiponectin levels.

By separating patients based on their BAT status, our data analyses provided novel findings and insights. Expected differences in BAT activity at baseline hyperthyroidism were seen, establishing the reliability of our measurements. There was no significant disparity between adiponectin levels between both groups, suggesting that adiponectin did not have an obvious relationship with BAT.

The BAT-negative group (n = 13) showed similar changes in body composition, cholesterol levels, adiponectin levels and adiposity after treatment as compared to the total sample (n = 21). The increases in weight, fat and lipids along with adiponectin suggest a negative change in metabolic health following attainment of euthyroidism. Remarkably, these significant changes were completely lost in the BAT-positive group, implying the preservation of metabolic profile in BAT-activity. This peculiarity further extends into the predictors for adiponectin. FT4 was found to no longer correlate with adiponectin in the BAT-positive group. These data suggest that BAT activity might protect against some of the physiological and metabolic changes associated with the correction of thyrotoxicosis. We explored potential reasons for these fascinating findings. Firstly, these differences may be explained by adipose tissue activity. Adiponectin, being a hormone secreted by white adipocytes [6–10], is expected to increase with restoration of euthyroidism as WAT increases following a reduction in catabolic hyperthyroid state. The presence of BAT will mitigate or negate the increase in WAT [38], thus reducing the adiponectin secretion in proportion to the expected weight gain as thyroid hormones declines. Hence, BAT abrogates the relationship of adiponectin and thyroid hormones. In support of this, Weiner J. et al. had previously demonstrated that cold exposure led to the compensatory recruitment of progenitor white adipocytes for de novo differentiation into beige adipose tissue in mice with thyroid dysfunction and impaired BAT activity [39]. Applying this reasoning, when hyperthyroidism was corrected in the BAT-negative group, the absence of BAT activity may have resulted in a compensatory increase in WAT adiposity to maintain thermogenic homoeostasis in the patients, causing increased adiponectin production and metabolic changes in only the BAT-negative group. Lastly, Yoneshiro et al. suggested a mechanism involving protein metabolism, by establishing that active BAT can protect against metabolic disturbances via stimulating BCAA catabolism in hyperthyroid patients [40]. Applying these findings clinically, increasing the browning of WAT particularly among those with minimal pre-existing BAT may be potentially utilized as an innovative therapeutic mechanism against the metabolic disruptions that hyperthyroid patients face upon correction of hyperthyroidism.

Enigmatically, although thyroid dysfunction has been traditionally associated with the notion of insulin resistance [1,2], our data unexpectedly showed no significant changes in insulin and HOMA-IR values when hyperthyroidism was corrected. We considered if adiponectin levels could have contributed to this, and if adiponectin could serve as a predictor for impaired glucose metabolism in hyperthyroidism, but both our data and various studies proved otherwise [41–47]. Our data in Table 4 showed a lack of correlation between adiponectin and HOMA-IR values when treating for Graves’ disease, and the absent correlation was unaffected by BAT activity of the patients. Similar lack of findings has been reported by Chng et al., and a possible reason suggested was that changes in insulin resistance in a thyrotoxic state occur only in individuals with a predisposition to glucose intolerance [30]. Increases in insulin resistance were thus unlikely to take place in participants that were physically lean and fit. Our study primarily involved Chinese females with normal BMI, healthy and without a predisposition to glucose intolerance, and hence an absence of significant insulin and HOMA-IR changes can be expected. Noteworthily, our data in Figure 5 revealed that BAT-positive patients appear to have lower HOMA-IR scores after treatment despite starting with similar baseline scores. Although this difference between the HOMA-IR scores did not reach a statistically significant level, we believe that this could potentially be due to the small sample size of the subgroups. Chondronikola et al. also supports the functional role of BAT activation in increasing insulin sensitivity in humans [48]. This links back and further augments the finding of BAT-activity serving as a protective factor against metabolic changes in hyperthyroidism treatment.

In our study, there are some limitations. Firstly, in terms of study design, we hypothesized that TH elevation in the hyperthyroid state may induce BAT activity even at normal room temperature without any cold-exposure typically used to stimulate BAT. This might have caused our measured BAT activity to be lower compared to a standard cold-induced BAT activation [3]. However, it seems that a small amount of active BAT is capable of protecting against significant physiological and metabolic changes, and it would be interesting to examine the data in a cold-induced BAT activation scenario. We believe that our findings should remain valid even in a cold-induced BAT activation scenario. Although it is potentially possible for BAT-negative people to become BAT-positive when studied in a colder climate, a true BAT-status (based on PET/MR) transformation can only be done through the process of browning of WAT into beige fat. This process is mediated by the PRDM-16 gene, and requires chronic cold exposure [49]. In an acute cold-induced scenario, the BAT-status of our patients should remain unperturbed, which meant our results should be valid. Secondly, the small sample size in our study probably accounted for some statistically insignificant observations. An example is the lack of statistical significance in the post-treatment differences of mean HOMA-IR scores between BAT subgroups despite a suggestive trend seen in Figure 5 and literature. Finally, there was a rather disproportionate gender and ethnic ratio within the subjects in our study. Ethnicity can influence the relationship between weight changes and insulin resistance [50], and adipokines may also exhibit sexual dimorphism [51]. Hence, it is hard to consider and account for the effects of ethnicity and gender in our study. The conclusion from our results will merit further validation in larger cohorts to ascertain the relationships between TH and adiponectin, as well as the protection against adverse metabolic changes by the presence of active BAT.

## 5. Conclusions

In summary, our results are consistent with studies demonstrating lowered adiponectin levels in a thyrotoxic state, with FT4 acting as a predictor for serum adiponectin levels when correcting thyroid hormone excess. Our study suggests that active BAT appears to protect against the development of adverse metabolic changes in the transition from thyrotoxicosis to euthyroidism. This novel finding may indicate the need to augment BAT to combat the unfavourable metabolic changes while treating patients with Graves’ disease.

## Appendix: Glossary

**Table:**

Δ	changes from treatment
18 F-FDG	18 F-fluorodeoxyglucose
ATD	anti-thyroid drug
BAT	brown adipose tissue
BMI	body mass index
DBP	diastolic blood pressure
DSAT	deep subcutaneous adipose tissue
DXA	dual-energy X-ray absorptiometry
ET	euthyroid
FOX	fatty acid oxidation
FT3	free T3 (triiodothyronine)
FT4	free T4 (tetraiodothyronine or thyroxine)
HDL	high-density lipoprotein
HOMA-IR	homoeostatic model assessment for insulin resistance
HT	hyperthyroid
IRT	infrared thermography
LDL	low-density lipoprotein
MR	magnetic resonance
NEFA	non-esterified fatty acids
PET	positron-emission tomography
PPAR-α	peroxisome proliferator-activated receptor-α
RMR	resting metabolic rate
SAT	subcutaneous adipose tissue
sBAT	supraclavicular brown adipose tissue
SBP	systolic blood pressure
SEM	standard error of mean
sFF	supraclavicular fat fraction
SSAT	superficial subcutaneous adipose tissue
SUV	standardized uptake value
VAT	visceral adipose tissue
WAT	white adipose tissue
WBC	whole body calorimetry
